# Supplementary material for: Atherogenic Index of Plasma Predicts Obstructive Coronary Artery Disease in Patients with Stable Angina Pectoris
Source: Diagnostics (Basel). 2023 Oct 19;13(20):3249. doi: 10.3390/diagnostics13203249 (PMC10606625; doi:10.3390/diagnostics13203249)
Supplement: Supplementary file 1 [file diagnostics-13-03249-s001.zip › diagnostics-2602019-supplementary.pdf]

Table S1 Correlation of AIP and cholesterol values

Variable	r	P value
TG mg/dl	0,85	< 0.000
HDL-C mg/dl	-0,71	<0,0001
LDL-C mg/dl	0.1	0.06

r: correlation coefficient; Other abbreviations as in Table 1.
